# Synchrotron radiation FTIR microspectroscopy enables measuring dynamic cell identity patterning during human 3D differentiation

**DOI:** 10.3389/fcell.2025.1569187

**Published:** 2025-05-21

**Authors:** Tanja Dučić, Francisco Rodriguez-Yañez, Elena Gonzalez-Muñoz

**Affiliations:** ^1^ ALBA Synchrotron Light Source, Barcelona, Spain; ^2^ Instituto de Investigacion Biomedica de Malaga y Plataforma en Nanomedicina-IBIMA Plataforma BIONAND, Malaga, Spain; ^3^ Department of Cell Biology, Genetics and Physiology, Universidad de Malaga, Malaga, Spain

**Keywords:** SR-FTIR, biomolecular conformation, IPSC, 3D embryoid bodies, neural spheroids, human morphogenesis, cell identity

## Abstract

Human cell fate specification, particularly in neural development, is difficult to study due to limited access to embryonic tissues and differences from animal models. Human induced pluripotent stem cells (hiPSCs) and 3D organoid models enable the study of early human neural development, surpassing limitations of 2D cultures by incorporating crucial cell-cell and cell-matrix interactions. In this study, we used synchrotron radiation-based Fourier transform infrared (SR-FTIR) microspectroscopy to examine biomolecular profiles of 3D-differentiated organoids, specifically embryoid bodies (EBs) and neural spheroids (NS), derived from hiPSCs. SR-FTIR allowed us to analyze these organoids’ cellular identity at a biomolecular level, offering a holistic view that complements specific cell markers. Our findings reveal distinct biomolecular identities in 3D organoids, with differences in DNA structure, lipid saturation, phospholipid composition, and protein conformations. This approach highlights that cellular identity is shaped by more than gene expression alone; it involves unique biomolecular compositions that can be detected even in complex, multicellular environments. By demonstrating the role of molecular configuration in cell differentiation, our findings suggest that differentiation processes extend beyond genetics, involving interdependent biochemical signals. This study demonstrates the unique efficacy SR-FTIR in analyzing human-specific 3D models for investigating complex multicellular differentiation mechanisms, offering new avenues for understanding the biochemical basis of human development and disease.

## 1 Introduction

The variety of mechanisms involved in human development during early cell fate specification has only recently begun to be elucidated due the relative inaccessibility of human embryo tissues and biological differences with animal models. This is specially challenging in brain development that requires an elaborate succession of cellular events that, when disrupted, can lead to neuropsychiatric disease which present great difficulty in studying and understanding their origin and possible therapeutic alternatives.

Neural specification of human induced pluripotent stem cells (hiPSCs), offers unprecedented opportunities for studying human neural development (Avior et al., 2016). The recent development of 3D neural cultures derived from hiPSCs offers a promising approach to understanding human brain development and disease (Pasca, 2018), surpassing the lack of cell-cell/cell-matrix interactions in monolayer cultures.

Our understanding has improved recently thanks to techniques allowing single-cell resolution in studying cell states (Hagey et al., 2016). Nevertheless, cell commitment during development has mainly been investigated in isolation at the population level by analyzing the effects of individual factors or, when using non-targeted approaches, the analysis is focused on cell expression profiles based on specific gene or protein identity markers (Jovic et al., 2022). This approach is not ideal due to the interdependent nature of the processes and the significant cellular transformations involved.

Although the use of cell identity markers is very useful and allows the analysis of specific cell populations, their use is not free of controversy regarding their fidelity or causality in determining cell identity, and it is becoming increasingly necessary to understand cellular transformation as a whole in order to achieve a deep understanding of the specification process during development and the factors that constitute the identity of a cell (McKinley et al., 2020).

Fourier transform infrared (FTIR) microspectroscopy is a highly effective analytical method used to examine various cellular components, including polysaccharides, nucleic acids, proteins, and lipids at the subcellular level. It leverages the natural ability of molecular systems to vibrate in resonance with different infrared light frequencies, creating unique spectra for each component (Miller and Dumas, 2010). Synchrotron radiation (SR)-FTIR microspectroscopy offers exceptional spectral resolution at the single-cell level, enabling the detailed analysis of cellular diversity within complex systems. Its micrometer-scale resolution, allows for monitoring the overall biochemical makeup of bulk samples (spanning tens of cubic microns). This method can detect subtle shifts in the composition of biological macromolecules, making it a valuable tool across various biological research fields for studying changes in chemical structure and conformation (Dumas, 2020).

The high spectral clarity and stable, fast measurements provided by synchrotron source FTIR (SR-FTIR) make it ideal for accurately resolving organic compounds, especially when compared to conventional globar sources. SR-FTIR spectroscopy provides approximately 1000 times the brightness of standard sources, enhancing the signal-to-noise ratio and enabling rapid acquisition of thousands of spectra over large sample areas with high peak precision (Baker et al., 2014). Additionally, SR-FTIR combines the high spatial resolution required to reach diffraction limits with excellent spectral detail, linking specific chemical groups to their vibrational peaks in IR spectra—thus aiding in the study of chemical properties in biological molecules and complexes.

Mid-infrared (IR) spectroscopy offers high sensitivity for analyzing the structure and conformation of proteins, lipids status, and nucleic acids, as well as complex biological substances like body fluids, cell cultures and tissues, often used in biomarker research (reviewed in (Dumas, 2020; Malek and Bambery, 2014; Finlayson et al., 2019)). Infrared spectroscopy is a crucial tool in “functional biology,” revealing extensive information from FTIR spectra, such as protein secondary structures through Amide I (1,600–1,700 cm^−1^) and Amide II (1,500–1,060 cm^−1^) bands, which reflect peptide backbone vibrations. These bands are sensitive to structural differences in proteins, such as α-helices, β-sheets, turns, and random coils, as influenced by hydrogen bonding environments. Beyond conformational analysis of proteins, FTIR provides detailed biochemical insights into samples, with prominent absorption patterns from lipids (2,800–3,020 cm^−1^), carbonyl (C=O) groups in lipids and proteins (1,480–1,780 cm^−1^), and nucleic acids (900–1,480 cm^−1^), which involve PO_2_^−^ stretching vibrations.

FTIR spectroscopy has broad applications beyond biological research and diagnostics, including material science, extreme environmental studies, archaeology, and planetary sciences. It is also pivotal in forensic science, as the biochemical makeup of fingerprints provides essential evidence. As a non-destructive technique sensitive to biomolecular conformational changes, FTIR microspectroscopy has been used to study mouse and rat healthy or injured brain tissue and there is still a limited number of studies using human brain tissue that confirm the capacity of this technology to be used as a potential diagnostic tool for disease mainly focused in brain tumors (Boseley et al., 2024; Liao et al., 2013; Steiner et al., 2023). However, one limitation in advancing neuroscience research lies, on one hand, in the relative difficulty of accessing human neural tissue samples, especially from pathologies where surgical interventions are highly unlikely, and on the other, in the challenge of tracking early stages of diseases or understanding the developmental characteristics of the nervous system itself. To address this issue, differentiated cell models derived from induced pluripotent stem cells provide a valuable alternative to animal models, allowing for the study of unique human-specific traits. The application of FTIR microspectroscopy in studying cell models derived from pluripotent cells remains limited and has largely focused on homotypic differentiations in 2D cultures (Ami et al., 2008; Heraud et al., 2010; Cao et al., 2014). Expanding its use to more complex systems, such as 3D cultures or *in vivo* models, could provide deeper insights into cellular differentiation and the dynamic biochemical changes that occur during development. While few research has explored its use in analyzing 3D structures, this has mainly focused on tumor organoids (Sun et al., 2024; Shao et al., 2024), with little information available regarding its use in understanding complex 3D multicellular differentiation. Such studies will not only enhance our understanding of the molecular features of this intricate process but also help identify potential abnormalities associated with these tissues.

In this study, we utilized both spontaneous and directed 3D differentiation of iPS cells: spontaneous differentiation to generate embryoid bodies (EBs) containing cell derivatives from the three germ layers, and directed differentiation formed neural spheroids with characteristic glial and neuronal profiles. We applied SR-FTIR technology to analyze cellular identity in terms of the composition and conformation of key/fundamental biomolecules, providing a holistic perspective that complements specific cell-type markers. Our findings reveal that 3D organoids exhibit notable differences in their biomolecular profiles, and conformational changes reflect a distinct macromolecular identity, including unique characteristics in DNA backbone structure, phospholipid abundance, lipid unsaturation levels, and predominant protein structures.

## 2 Methods

### 2.1 Cell culture and 3D spheres generation

We used previously generated iPS cell lines from female donors (3 cell lines) with clearances from the bioethical committee and Review Board of the Spanish National Research Ethics Service (#PR-03-2018). They were cultured in hES medium on mitomycin-C-treated mouse fibroblasts as previously described (Gonzalez-Munoz et al., 2014; Lopez-Caraballo et al., 2020a; Lopez-Caraballo et al., 2020b). For 3D neural spheroid (NS) generation we used already published protocol (Marton et al., 2019) with some modifications. iPSCs were dissociated and plated in low-attachment 96-well U-bottom plates in mTeSR1 medium with ROCK inhibitor Y-27632. Spheroids were differentiated into neuroectodermal lineage using SMAD inhibitors (and maintained in differentiation medium supplemented with growth factors (EGF, bFGF) and SHH agonist. From day 25, NS were cultured with T3, biotin and neurotrophic factors (NT-3, BDNF), to promote maturation. For embryoid body (EB) formation, iPSCs were aggregated in AggreWell-800™ and cultured in hES medium without bFGF as previously described (Gonzalez-Munoz et al., 2014; Lopez-Caraballo et al., 2020a).

At day 40, three dimensional NS and EB were either used for RNA isolation or fixed using 4% paraformaldehyde incubation for 20 min at 25°C. Data correspond to the average of 3 independent differentiation experiments done in triplicate (EB) or quadruplicate (NS) from three different iPSC clones (n = 9 for EB and n = 12 for NS).

### 2.2 Immunofluorescence

NS and EB were fixed in 4% paraformaldehyde, cryoprotected in 30% sucrose, and embedded in OCT compound. Cryosections were blocked and incubated with primary and secondary antibodies, counterstained with Hoechst 33258, and imaged using a Leica SP5 II confocal system. Fluorescence intensity was quantified using Fiji software (Shihan et al., 2021; Schindelin et al., 2012).

### 2.3 Quantitative PCR

RNA was isolated using the NucleoSpin RNA Mini kit, and cDNA was synthesized using SuperScript II SuperMix. qPCR was performed with SYBR Green on a CFX96 Real-Time System, using actin, gapdh, and tbp as reference genes (Supplementary Table S4).

### 2.4 SR-FTIR measurements and analysis

Samples were analyzed at the MIRAS beamline (ALBA Synchrotron) using a Hyperion 3,000 microscope. Spectra were collected in transmission mode (10 μm × 10 µm aperture) across the 4,000–900 cm^−1^ range. Principal component analysis (PCA) was performed using Orange software (Demšar et al., 2013; Toplak et al., 2017; Toplak et al., 2021).

## 3 Results

### 3.1 Three-dimensional organoids derived from the spontaneous and directed differentiation of induced pluripotent stem cells (iPSCs) exhibit characteristic molecular markers of specific trilineage and neural differentiation

Embryoid bodies (EBs) were generated as 3D trilineage differentiation organoids by allowing iPSCs to spontaneously differentiate in suspension cultures without growth factors, promoting simultaneous and spontaneous differentiation into cell types representing the three germ layers: endoderm, mesoderm, and ectoderm. Neural spheroids (NS) were produced through a modified protocol (Marton et al., 2019) that included inhibiting the SHH signaling pathway after neuronal induction. This approach activates oligodendrogenesis, generating therefore the three main neural cell types of the central nervous system—neurons, astrocytes, and oligodendrocytes—that co-develop both spatially and temporally, mirroring endogenous cellular diversity.

**FIGURE 1:**
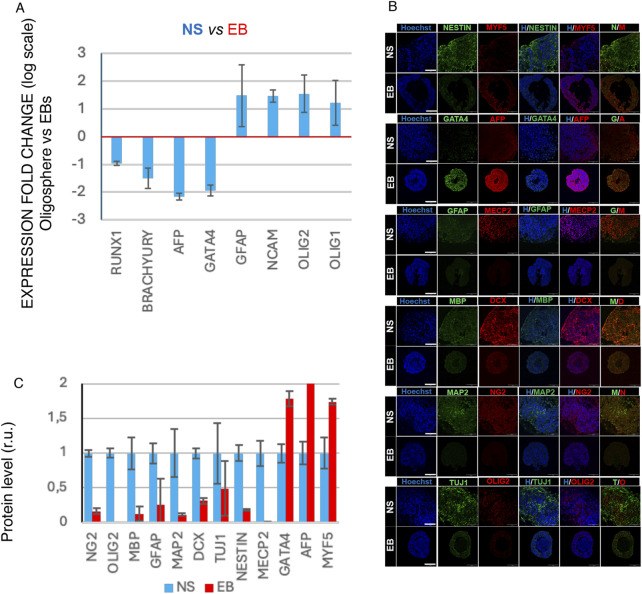
Cell marker characterization of NS and EB 3D organoids differentiated from human iPSC lines. **(A)** qRT-PCR data showing regulation of differentiation markers *GATA4* and *AFP* (endoderm), BRACHYURY and *RUNX1* (Mesoderm), and for ectoderm and neural differentiation, *NCAM* for neurons, *GFAP* for astrocytes, and *OLIG1* and *OLIG2* for oligodendrocytes at day 40 of either *in vitro* differentiation. Average folding change expression values ± STD (relative to spontaneous differentiated EB) are represented (logarithmic scale). **(B)** Representative immunofluorescence analysis image of markers of mesoderm (MYF5), GATA4 and AFP for endoderm, NG2 and OLIG2 for oligodendrocyte progenitors, MBP for oligodendrocytes, GFAP for astrocytes, and MAP2, DCX, TUJ1, NESTIN, and MECP2 for neuronal progenitors and early neurons (scale bar 100 µm). **(C)** Protein level quantification based on relative fluorescence intensity.

After 6 weeks, RNA from NS and EBs was extracted for quantitative PCR analysis targeting cell-type markers: *AFP* and *GATA4* for endoderm, *BRACHYURY* and *RUNX1* for mesoderm, and for ectoderm and neural differentiation, *NCAM* for neurons, *GFAP* for astrocytes, and *OLIG1* and *OLIG2* for oligodendrocytes (Figure 1A). Additionally, NS and EBs were fixed, cryosectioned, and immunostained to detect proteins associated with specific cell identities, including MYF5 for mesoderm, GATA4 and AFP for endoderm, NG2 and OLIG2 for oligodendrocyte progenitors, MBP for oligodendrocytes, GFAP for astrocytes, and MAP2, DCX, TUJ1, NESTIN, and MECP2 for neuronal progenitors and early neurons (Figure 1B).

Quantitative PCR and immunofluorescence data, supported by fluorescence intensity quantification (Figure 1C), reveal significant differential marker expression aligned with trilineage identities in EBs and neural cell types in NS. These data confirm the reliability of the 3D model in accurately reflecting the distinctive co-differentiation processes.

### 3.2 SR-FTIR spectroscopy enables the identification of complex differentiated 3D structures based on characteristic biomolecular profiles that reflect their interconnected cellular identities

**FIGURE 2:**
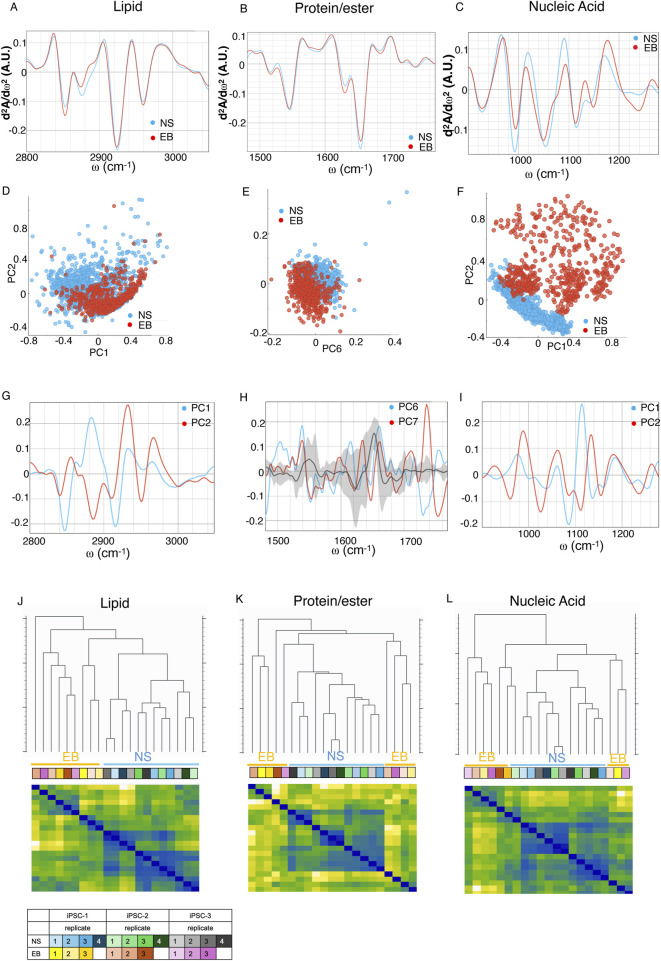
Second derivative of the FTIR averaged spectra of fixed neural spheroids (NS) (blue) and trilineage (EB) (red) differentiated iPSC in the **(A)** lipids’ spectral region of 3,020–2,800 cm^−1^, **(B)** proteins and carbonyl area, 1,800–1,480 cm^−1^, and **(C)** the nucleic acids region, 1,200–900 cm^−1^. Graphs in **(D,F,H)** represent the PCA analysis and values of the PC scores for each region assigned above; graphs **(E,G,I)** show the contribution of individual absorbance to the PCAs (loading values) of the specified principal components in blue and red. Black line and grey shadow represents the average and SD contribution of all principal components explaining 80% of the variance. **(J–L)** Hierarchical clustering (top dendrograms) and leveraged heatmaps (bottom matrices) of NS and EB organoids, based on the average FTIR absorption spectra of each biological replicate. Clustering was performed using Euclidean distances calculated across three distinct spectral regions: lipid-associated bands **(J)**, protein and carbonyl-associated bands **(K)**, and nucleic acid-associated bands **(L)**. In the heatmaps, warmer colors indicate greater similarity (lower distance) between replicates, while cooler colors indicate greater dissimilarity. This visualization allows grouping of biologically related samples based on their biochemical spectral profiles. The color-coded table indicates the identity and grouping of the biological replicates. Data represent independent differentiation experiments performed in triplicate for EBs and quadruplicate for NSs, derived from three different iPSC clones (n = 9 for EB and n = 12 for NS).

EBs and NS cryosections were analyzed using SR FTIR microspectroscopy, revealing primary absorption features of key biomolecules: proteins (Amide I and II: 1,480–1,700 cm^−1^), lipids (2,800–3,000 cm^−1^) carbonyl (C=O) groups, and nucleic acids and sugars that predominantly absorb between 1,000 and 1,500 cm^−1^ (Dumas, 2020) (Supplementary Figure S1). In Figure 2, the second-derivative spectra show the lipid region (A), Amides I and II and carboxyl group (B), and nucleic acids (C), highlighting distinctive spectral variations between EB and NS samples across all regions (Figures 2A–C), confirmed by PCA grouping of these sample types (Figures 2D–F; Supplementary Table S2).

In the lipid region, the differences are evident in bands at ∼2,850, ∼2,874, ∼2,925, and ∼2,960 cm^−1^, corresponding to ***v***_s_CH_2_, ***v***_s_CH_3_, ***v***_as_CH_2_ and ***v***_as_CH_3_ respectively (Loutherback et al., 2016), along with the ∼3,010 cm^−1^ peak (***v*** (*H−C =* ), indicating fatty acid unsaturation (Figure 2A; Supplementary Table S1). The PCA analysis further substantiates these distinctions, as these bands significantly contribute to the separation of the EB and NS groups (Figures 2D,G; Supplementary Table S2). For the protein/ester region, variations primarily involve the Amide I and II band positions (Figure 2B; Supplementary Table S1), where PCA loadings indicate shifts in secondary protein structures, notably around ∼1,620, ∼1,650, and ∼1,680 cm^−1^, representing intramolecular β-sheet, α-helix, and turn and loops structures, respectively, alongside the ∼1,740 cm^−1^ carbonyl peak from phospholipid acyl chains or carbonylated proteins (Loutherback et al., 2016) (Figures 2E,H; Supplementary Table S2). Within Amide II, differences emerge at ∼1,545 to 1,555 cm^−1^ for α-helix and random coil structures (Malek and Bambery, 2014; Dučić et al., 2022) and at intramolecular β-sheet structure peak near ∼1,570 cm^−1^ (Figure 2B; Supplementary Table S2).

In the nucleic acid and carbohydrate absorbtion region (1,200–900 cm^−1^), multiple bands show differences (Figure 2C; Supplementary Table S1). PCA indicates distinct vibrational band contributions at ∼975, ∼1,027, ∼1,060 cm^−1^, corresponding to DNA, Z-form DNA, with the latter particularly enhanced in Z-form DNA (Zhang et al., 2016). Shifts at ∼1,110–1,115 cm^−1^ and ∼1,130–1,135 cm^−1^ relate to DNA methylation (Li et al., 2018) and RNA (Gioacchini et al., 2014), respectively (Figures 2F,I; Supplementary Table S2), suggesting notable DNA reorganization and epigenetic variation based on organoid type (Knaupp et al., 2017; Li et al., 2021).

Euclidean distances were calculated for each spectral region’s averages to create a hierarchical clustering of the different samples (Figures 2J–L), that derived from distinct experimental replicates from three different iPSCs to ensure robustness and reproducibility of the results. We found differential segregation of samples within each spectral region according to their differentiation group, confirming that the molecular configuration profile based on their spectral features varies and is therefore definitive of cellular identity within the organoids.

**FIGURE 3:**
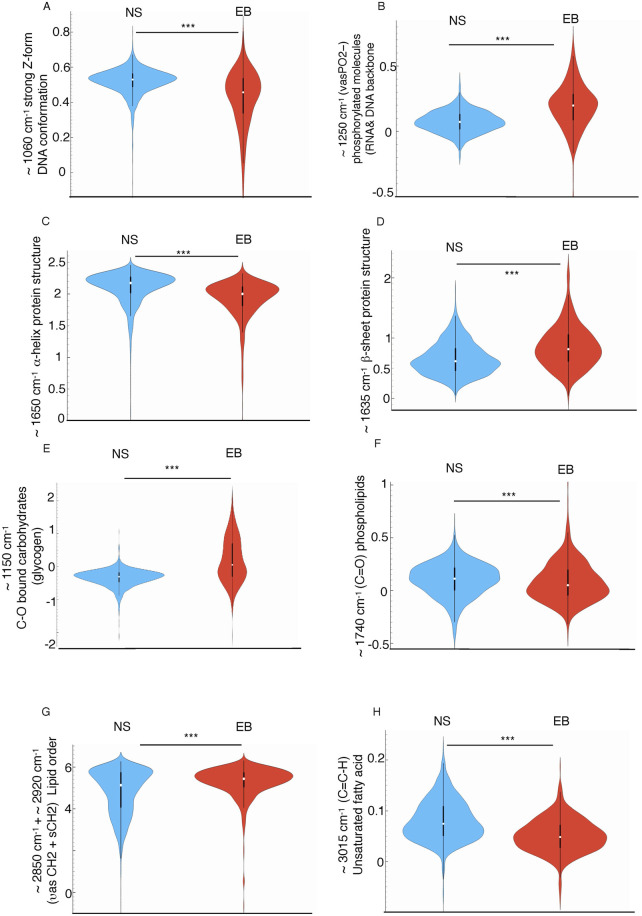
Violin plots showing the normalized mean integral area values of second derivative average spectra of NS and EB organoids at selected spectral regions: **(A)** ∼1,060 cm^-1^ assigned to strongly enhanced Z-form DNA **(B)** ∼1,250 cm^-1^ assigned to (νasPO^2−^) related to DNA backbone conformation **(C)** ∼1,650 cm^-1^ assigned to α-helix protein conformation, **(D)** ∼1,635 cm^-1^ related to β-sheet protein conformation, **(E)** ∼1,150 cm^-1^ corresponding to −CO− stretching related to glycogen, **(F)** ∼1,740 cm^-1^ assigned to the ester carbonyl groups (vC = O) related to phospholipids, **(G)** (∼2,850 + 2,920 cm^−1^) sum of asymmetric and symmetric CH2 picks area related to the total amount of lipid present in the cells, **(H)** ∼3,010 cm^-1^ assigned to unsaturated fatty acids. Values were calculated from unit vector-normalized spectra following baseline correction and second derivative transformation. Integration was performed using the “integral from 0” method within Orange Spectroscopy software (ANOVA test ***p < 0.005). The y-axis represents unitless integral area values, enabling relative comparisons across groups. These measurements reflect the abundance of specific molecular features between NS and EB populations.

These differences were supported by the analysis of the integral area of the specific peaks, indicative of abundance of specific biomolecule and conformation (shown in Figure 3; Supplementary Table S2). For DNA backbone conformation, peak areas around ∼990 and ∼1,060 cm^−1^, associated with DNA-backbone and Z-form DNA that has been associated with histone acetylation and epigenetic modifications (Zhang et al., 2016; Gioacchini et al., 2014; Beknazarov et al., 2020), were statistically higher in the NS group, while ∼1,150 and ∼1,250 cm^−1^ peaks, indicative of different C-O bound carbohydrates (glycogen) (Malek and Bambery, 2014; Heraud et al., 2010) content and RNA/DNA structures respectively (Martinez-Rovira et al., 2020), were more prominent in EB organoids. Although most molecular events during cell specification have traditionally been studied in the context of specific transcriptional regulator activity, the epigenetic roles associated with nucleic acid conformational changes and molecular bonding remain underexplored. Z-DNA has gained attention for its potential role in transcriptional regulation; it is thought to form in the promoter region within open chromatin, induced by chromatin remodeling factors, and to stabilize this open structure through binding with proteins such as ADAR1 or Nrf2, thereby facilitating downstream transcriptional events (Liu et al., 2001; Maruyama et al., 2013). The distinct DNA conformations observed between EB and NS organoids suggest a potential relationship between these conformational changes and the involvement of specific proteins, either as causative factors or as a consequence of these changes. This includes the possible roles of histone acetyltransferases and Z-DNA binding proteins, such as mentioned ADAR1, Nrf2, or DLM-1 (Kim et al., 2018) for cell fate decisions. While the identification of Z-DNA is based on previously reported vibrational signatures in the ∼1,060 cm^−1^ region, we acknowledge that this remains a putative spectral assignment. Nonetheless, experimental studies using isotopically labeled oligonucleotides and induced B-to-Z transitions have provided direct evidence linking these IR bands to the Z-DNA conformation (Banyay et al., 2003; Duan et al., 2023). *In vivo* formation of Z-DNA under histone acetilation conditions (Zhang et al., 2016) or after histone remodeling complex overexpression (Li et al., 2020), further supports its functional relevance. However, complementary structural approaches such as NMR or crystallography would be required to definitively confirm its presence in our system.

Additionally, the ∼1,070 cm^−1^ peak area, representing −CO−O−C stretching in cholesterol esters and phospholipids, was elevated in EBs *versus* NS (Supplementary Figure S1G).

In the protein region, significant differences were found in Amide I and II integral areas (Supplementary Table S2), indicating changes in protein quantity, structure, or function. Protein conformation variations were also observed in peak areas near ∼1,650 cm^−1^, ∼1,645 cm^−1^, and ∼1,635 cm^−1^, attributed to α-helix, random coil, and β-sheet structures, respectively (Pezolet et al., 1992; Wellner et al., 1996; Sarroukh et al., 2011; Bhatia et al., 2015; SAJ and Joye, 2020) (Figures 3C,D; Supplementary Table S2). Notably, NS samples exhibited a higher presence of α-helix structures, while EBs had more β-sheet structures. The β-sheet/α-helix ratio is linked to proteins’ responses to mechanical stimuli (Rustem et al., 2012), presenting a potential avenue for examining the connection between mechanical influences, recently described as regulators of morphogenetic events (Goodwin and Nelson, 2021), and the underlying molecular mechanisms that involve changes in the composition and conformation of biomolecules involved in this regulation.

Although part of this difference likely reflects the distinct proteomic profiles of each organoid type, as also indicated by marker expression patterns (Figures 1A,C), it may also result from conformational shifts driven by mechanobiological context. Mechanical forces are known to regulate protein folding and aggregation states, often without altering gene expression, by influencing the energy landscape of protein structure (Discher et al., 2009; Vining and Mooney, 2017). Thus, the β-sheet/α-helix ratio observed here may represent an integrated biochemical signature of both protein composition and mechanotransductive regulation, reinforcing the value of SR-FTIR in capturing subtle, functionally relevant structural properties in differentiating cells.

In the lipid region, a significant increase in methylene groups (both ***v***_s_CH_2_ and ***v***_as_CH_2_) was observed during trilineage development compared to neural differentiation (Figures 3G,H; Supplementary Table S2). This increase suggests longer acyl chains, impacting membrane rigidity, and an overall rise in lipid content (Malek and Bambery, 2014) within these cells as confirmed by the higher cumulative methylene and methyl peak areas in EBs (Supplementary Table S2). Additionally, the significant increase in the ∼3,010 cm^−1^ peak area (Figures 3G,H; Supplementary Table S2) —associated with fatty acid unsaturation—in NS, alongside the elevated symmetric/asymmetric methylene stretching ratio (***v***_s_CH_2_/***v***_as_CH_2_) in EBs (linked to membrane rigidity) (Sandt et al., 2013), aligns with the observation of greater membrane lipid fluidity during neural differentiation compared to trilineage differentiation.

Meanwhile, other lipid structural features, such as the abundance of ester carbonyl group stretching vibrations (***v*** C=O), associated with membrane permeability, and the ratio of this peak to the sum of methylene and methyl band areas (***v*** CH_2_ and ***v*** CH_3_), which has been correlated with lipid peroxidation (Dučić et al., 2019; Mendelsohn et al., 2006; Kreuzer et al., 2020), remain similar across both trilineage and neural differentiation. These findings indicate that lipid composition and structural variations are not generalized but rather specifically targeted to certain aspects, such as membrane fluidity. These distinctions suggest that particular structural and functional adaptations in lipid membranes may underpin mechanisms or functional outcomes tied to the complex multicellular differentiation processes.

## 4 Discussion

Growing evidence implicates metabolic pathways as central regulators of cell fate and function, suggesting that these pathways not only supply energy for cellular activities but also shape and define cellular identity (Ghosh-Choudhary et al., 2020), influencing the epigenetic regulation of gene expression (Tatapudy et al., 2017). For instance, nicotinamide N-methyltransferase (NNMT) is a key enzyme in metabolic regulation, essential for establishing and maintaining the histone H3 repressive mark (H3K27me3) during pluripotency (Sperber et al., 2015). NNMT is particularly abundant in adipose tissue and the liver, where its expression impacts lipid accumulation during cell differentiation processes (Komatsu et al., 2018; Xu et al., 2022), highlighting the interconnection between cell identity and biomolecular composition. In the context of neural stem cells, glucose and lipid metabolism play critical roles in processes such as proliferation, differentiation, and quiescence, with several studies emphasizing the importance of lipid accumulation in human neurogenesis (reviewed in (Angelopoulos et al., 2022)). Our data add a new perspective to this connection, highlighting the qualitative profile of these macromolecules as part of a coordinated process during multicellular co-differentiation, suggesting their active role in this process.

Our data provide highly relevant insights into early cellular development and specification, shedding light on the critical role that biomolecular composition and conformation play in determining cellular identity. The cellular macromolecular profile can be detected not only in homogeneous cell populations (Ami et al., 2008; Heraud et al., 2010; Cao et al., 2014; Dučić et al., 2019; Sandt et al., 2012; Dučić et al., 2023), but even in extremely complex cellular environments like those used here, which involve the simultaneous and cooperative emergence of various cell types. These findings suggest that differentiation and specification extend beyond a complex gene expression regulatory network; they are closely linked to the biomolecular composition and structure of cells during differentiation, which is therefore tightly related to cellular identity. While our data do not clarify the direction of causality between molecular configuration and gene expression profiles, they certainly establish a connection and add a new layer of complexity to cellular identity, simultaneously providing a novel tool for its definition. Furthermore, they allow for compelling hypotheses in which molecular composition and configuration underlie the intercellular regulation occurring during multicellular differentiation.

Differential specification regulation in interacting cells remains a key topic in human developmental biology. Most approaches have focused on the role of morphogens and transcriptional regulation via ligands and receptors, progressively contributing to the transcriptional restriction that leads to cellular identity. Recently, however, attention has shifted to how mechanical forces shape developing tissues and to the physical mechanisms of morphogenesis, leveraging biophysical measurements (Goodwin and Nelson, 2021; Mammoto et al., 2013). Developing tissues are subject to both intrinsic mechanical signals from active forces and changes in tissue mechanical properties, as well as extrinsic mechanical signals, such as constraint, compression, pressure, and shear forces.

It is essential to highlight that mechanical forces also impact another key developmental aspect—cellular differentiation. Physical forces can activate intracellular signaling cascades or modify nuclear envelope mechanics, influencing the activation or nuclear localization of mechanosensitive transcription factors, which then lead to downstream changes in gene expression, altering cell behavior and fate (Vining and Mooney, 2017; Hampoelz and Lecuit, 2011; Kumar et al., 2017). Such nuclear-level changes could indirectly impact tissue material properties, ultimately resulting in morphological changes.

While the study provides robust evidence of group-specific spectral differences supported by multivariate analyses, it is important to acknowledge certain limitations. The number of biological replicates per group is modest and may limit the generalizability of the findings. Although each replicate was independently generated and analyzed, increasing the sample size in future studies would enhance statistical power and capture broader biological variability. Additionally, our conclusions are based on exploratory, unsupervised methods (e.g., hierarchical clustering and PCA) rather than predictive modeling. While this avoids risks of overfitting, it also limits the formal assessment of classification accuracy. Future work may benefit from combining larger replicate numbers with supervised approaches to validate and extend the current findings.

Furthermore, all cell lines used in this study were derived from female donors. Although no consistent sex-dependent effects have been reported in spontaneous or directed neural differentiation under standard culture conditions, subtle sex-linked variability in gene regulation, chromatin structure, has been described (Sugathan and Waxman, 2013; Ober et al., 2008). Importantly, recent studies have shown both no significant sex-related differences in early neural induction from human pluripotent stem cells (Strano et al., 2020) or limited effect on the expression of specific genes (Pottmeier et al., 2024), indicating minimal impact on the results presented here. Nonetheless, including cell lines from both sexes in future studies would strengthen the translational robustness of spectral phenotyping approaches.

Finally, although SR-FTIR spectroscopy provides spatial resolution in the range of ∼3–10 μm, enabling single-cell level analysis (Dučić et al., 2022; Dučić et al., 2023; Andjus et al., 2019; Pascolo et al., 2014; Hackett et al., 2013; Doherty et al., 2016) this resolution is approximate and may integrate signals from adjacent cells in densely packed 3D structures such as organoids or embryoid bodies. Furthermore, the use of spectral averaging across many cells, while improving the signal-to-noise ratio, may mask the presence of rare subpopulations or transitional states. Although consistent spectral segregation between groups was observed, a more detailed exploration of cellular heterogeneity would benefit from increasing sampling density or combining SR-FTIR with higher-resolution or complementary single-cell techniques (Heraud et al., 2010; Wang et al., 2021).

Our approach employs a human model based on pluripotent stem cells 3D differentiation, proving to be extremely valuable for studying specific developmental characteristics of the nervous system, often not replicated in animal models (Walsh et al., 2024; Li et al., 2023). This model also enables simultaneous and comparative analysis of different experimentally controlled differentiation patterns.

Our findings indicate that biomolecular composition and configuration are also crucial factors during early developmental stages—an aspect that remains largely unexplored. Further studies are needed to establish causal relationships between genetic developmental patterns, the influence of mechanical forces, and molecular composition and structure. While the first two are beginning to be elucidated (Vining and Mooney, 2017; Hampoelz and Lecuit, 2011; Kumar et al., 2017), the role of molecular composition and conformation in this process remains uncharted. This study, therefore, serves as evidence of its significant role in human development.

## 5 Conclusion

This research underscores the value of SR-FTIR microspectroscopy for high-resolution biomolecular profiling in trilineage and neural hPSC-derived organoids, providing insights into cellular identity in 3D differentiation. The distinct biochemical and structural profiles observed among organoids reveal that cellular identity extends beyond traditional gene expression markers, implicating specific biomolecular composition and conformation besides mechanical forces in cell fate decisions. The study highlights SR-FTIR as a promising tool for investigating human developmental biology and understanding potential disease mechanisms that arise from differential and potentially aberrant cellular differentiation.

## Data Availability

The original contributions presented in the study are included in the article/Supplementary Material, further inquiries can be directed to the corresponding author.
