# Supplementary material for: Synchrotron radiation FTIR microspectroscopy enables measuring dynamic cell identity patterning during human 3D differentiation
Source: Front Cell Dev Biol. 2025 May 21;13:1569187. doi: 10.3389/fcell.2025.1569187 (PMC12133876; doi:10.3389/fcell.2025.1569187)
Supplement: Supplementary file 1 [file DataSheet1.pdf]

Supplementary Material

Synchrotron Radiation FTIR microspectroscopy enables measuring dynamic cell identity patterning during human 3D differentiation

Tanja Dučić[1], Francisco Rodriguez-Yañez[2] and Elena Gonzalez-Muñoz[2,3*]

[1] ALBA Synchrotron light source, Carrer de la Llum 2-26, 08290 Cerdanyola del Vallès, Barcelona, Spain

[2] Instituto de Investigacion Biomedica de Malaga y Plataforma en Nanomedicina-IBIMA Plataforma BIONAND, Malaga, Spain

[3] Dept. Cell Biology, Genetics and Physiology, Universidad de Malaga, Malaga, Spain. egonmu@uma.es

1 Supplementary Method Data

CELL CULTURE AND 3D SPHERES GENERATION

We used previously generated iPS cell lines from female donors (3 cell lines) with clearances from the bioethical committee and Review Board of the Spanish National Research Ethics Service (#PR-03-2018) [1]. They were cultured in standard human pluripotent cell culture medium (hES medium) consisting in DMEM/F12 containing 20%KSR (Knock Out Serum Replacement, Gibco), 10ng/ml of human recombinant basic fibroblast growth factor (bFGF, Miltenyi Biotec 130-093-838), NEAA (1:100, Thermo Fisher Scientific, 11140076), 1xL-Glutamine (1:100, Thermo Fisher Scientific, 25030081), 5.5mM β-mercaptoethanol (Sigma Aldrich, M3148), penicillin and streptomycin (1:100, Thermo Fisher Scientific, 15140122). iPS cells were cultured on top of mitomycin-C mouse fibroblasts and picked mechanically as previously described [1-3]. All cell lines were regularly tested for mycoplasma using PCR validation (Venor GeM Classic, Minerva Biolabs) and found to be negative.

For the generation of 3D neural spheroids (NS) we used already published protocol [4] with some modifications. hiPS cells were dissociated into single cells and 9,000 cells were plated in each well of a low attachment 96-well U-bottom (Corning, CLS7007) to obtain a uniformly-sized spheroid per well in mTeSR1 medium (Stem Cell Technologies, 05850) supplemented with the ROCK inhibitor Y-27632 (10 µM, StemCell Techonologies, 72307) and incubated at 37ºC with 5% CO_2. After 18–24 hours, spheroids consisting of approximately 10,000 cells were visible and subjected to media exchange in mTeSR1 medium supplemented with two SMAD pathway inhibitors– LDN-193189 (100nM, Miltenyi Biotech,130-106-540) and SB-431542 (10 µM, R&D systems, 1614) to force neuroectodermal differentiation.

On day 6 in suspension, the spheroids were transferred to differentiation and maintenance medium (DMM) containing DMEM/F12 (Thermo Fisher Scientific, 11330–057), B-27 Supplement without vitamin A (Thermo Fisher Scientific, 12587010), N2 Supplement (Thermo Fisher Scientific, 17502048), minimum essential media (MEM) Non-Essential Amino Acids (1:100, Thermo Fisher Scientific, 11140076), GlutaMax (1:100, Thermo Fisher Scientific, 35050079), human insulin (25 µg

ml^{-1}, Sigma-Aldrich, I9278-5ML), β-mercaptoethanol (0.1 mM; Sigma-Aldrich M3148), and penicillinstreptomycin (1:100, Thermo Fisher Scientific, 15140163). The DMM medium was supplemented with 20 ng/ml EGF (R&D Systems, 236-EG-01M) and 20 ng/ml basic fibroblast growth factor (bFGF) (Peprotech, 100–26) for 19 days (until day 24) with daily medium change in the first 10 days, and every other day for the subsequent 9 days. The small molecule SHH pathway smoothened agonist (SAG, 1 µM, Millipore Sigma, 566660) was added from day 12 to day 24. From day 25 to day 35, NS were cultured in DMM supplemented with T3 (60 ng/mL, Sigma Aldrich, T2877), biotin (100 ng/mL, Sigma Aldrich, B4639), NT-3 (20 ng/mL, Peprotech, 450–03), BDNF (20 ng/mL, Peprotech, 450–02), cAMP (1 µM, Sigma Aldrich, D0627), HGF (5 ng/mL, Peprotech, 315–23), IGF-1 (10 ng/mL, VWR, 100–11), and PDGF-AA (10 ng/mL, R&D Systems, 221-AA). From day 35 to day 40, NS were cultured in complete DMM (DMM supplemented with T3, biotin, cAMP, and ascorbic acid (AA, 20 µg/mL, Wako Pure Chemical, 323–44822).

Spontaneous trilineage differentiation well be performed using 3D embryoid body (EB) differentiation as previously described in our group [1, 2]. Briefly, hiPS cells were dissociated into single cells and plated into AggreWell-800™ to form 10,000 cells-spheres and transferred into ultra-low attachment plastic dishes (Thermo Fisher Scientific, 3262) as described above in hES medium with ROCK inhibitor Y-27632 (10 µM, StemCell Techonologies, 72307). After 48 hours, media was changed to hES medium without bFGF. From day 4 to day 40 EB were culture in hES medium without bFGF with medium exchange every 3 days.

At day 40, three dimensional NS and EB were either used for RNA isolation or fixed using 4% paraformaldehyde incubation for 20 minutes at 25ºC. Data correspond to the average of 3 independent differentiation experiments done in triplicate (EB) or quadruplicate (NS) from three different iPSC clones (n=9 for EB and n=12 for NS).

IMMUNOFLUORESCENCE

Three dimensional NS and EB were fixed sing 4% paraformaldehyde incubation for 20 minutes at 25ºC, transferred to 30% sucrose (Sigma Aldrich, S0389) for 24 hours and then into embedding medium (Tissue-Tek OCT Compound 4583, Sakura Finetek) and snap-frozen in an OCT-filled mold immersed in isopentane on a liquid nitrogen-cooled metal surface. Samples were stored at − 80 °C until 8–µm-thick cryosections were performed using a Leica cryostat and placed into CaF2 13x0.5mm slides (transparent for infrared and VIS light, Crystran UK) and glass coverslips (Electron Microscopy Sciences, 7229004) as recipient slides to undergo further analysis in ALBA by SR-FTIR beamline MIRAS and immunofluorescence assay, respectively.

For immunofluorescence, after rehydration with PBS, sections were blocked for 1 h at 25ºC in PBS containing 5% normal goat serum (NGS, Jackson ImmunoResearch) and 0,2% Triton X-100 (Sigma Aldrich, X100). The sections were then incubated overnight at 4°C with primary antibodies (Supplemetary Table S3) diluted in PBS containing 5% NGS and 0.2% Triton X-100. After three washes with PBS, sections were incubated with appropriate secondary antibodies (SI Table S3) for 1 h at room temperature. The slides were counterstained with Hoechst 33258 (Merck) and mounted in ProLong™ Diamond mounting media (Thermo Fisher). Fluorescence images were acquired using a Leica SP5 II confocal system. Quantification of each specific marker was done by calculating fluorescent intensity in stained sections using Fiji software [5, 6].

QUANTITATIVE PCR

mRNA was isolated using the NucleoSpin RNA Mini kit for RNA purification (Macherey-Nagel, 740984) according to manufacturer´s protocol and template cDNA was prepared by reverse transcription using the SuperScript II SuperMix for qRT–PCR (Thermo Fisher Scientific, 11752250). qPCR was performed using SYBR Green (Thermo Fisher Scientific, 4312704) on a CFX96 Real Time System (Bio-Rad C1000™ Thermal Cycler). *Actin, gapdh* and *tbp* were used as reference genes to normalize gene expression levels. Primers used are listed in Supplementary Table S4.

SR-FTIR MEASUREMENTS AND ANALYSIS

Samples were analyzed at the beamline MIRAS BL01 (ALBA Synchrotron, Spain), by using the 3000 Hyperion microscope coupled to a Vertex 70v spectrometer and a liquid nitrogen-cooled mercury cadmium telluride (MCT) detector. The spectroscopic data were collected in transmission mode using the 36x Schwarzschild objective/condenser and an aperture size of 10 μm x 10 μm. Between 60 and 120 cell spectra for each sample were collected in the 4000−900 cm^{-1} mid-infrared range at a spectral resolution of 4 cm^{-1} with 128 co-added scans per spectrum. Spectra were collected by using the OPUS software (version 8.2, Bruker Company).

Spectral analysis for every single cell and second derivative (Savitzky-Golay filter, 17 smoothing points, third polynomial order, and vector normalization), was implemented for three different areas: 3020−2800 cm^{-1} lipid area, 1800− 1480 cm^{-1} proteins and esters, and 1480−900 cm^{-1} nucleic acids and carbohydrate region. Vector normalization was applied after differentiation, as this normalization technique does not require a reference peak.

The principal component analysis (PCA) for each data set was performed after the second derivative calculation, and normalization of spectra. The PCA analysis was performed using the Orange software (Bioinformatics Laboratory of the University of Ljubljana [7], Version 3.37.0), with the spectroscopy package [8, 9](Version 1.10.2) on baseline corrected and unit vector normalized spectra, for spectra without second derivative transformation. The same software was also used to perform PCA analysis on the different wavenumber regions, to calculate integral area mean values of second derivative average spectra at specific wavelengths ranges using integral from 0 method and setting the lower and upper limits of integration at desired wavelengths and to build hierarchical clustering based on the calculation of Euclidean distances among groups, computing a matrix of pairwise distances between average spectra at selected regions where data are normalized to ensure equal treatment of individual features.

Supplementary Table S3. List of primary and secondary antibodies used for immunofluorescence assays

.

ANTIBODY	Source	Identifier
mouse anti Brachyury	Santa Cruz Biotechnology	sc-166962
mouse anti-Gata4	Santa Cruz Biotechnology	sc-25310
rabbit anti-Ng2	MILLIPORE	AB5320
rabbit anti Olig2	*Chemicon/Millipore*	*AB9610*
mouse anti Mbp	Cusabio	000061M0m
mouse anti Gfap	DAKO	M076101-2
mouse anti Tuj1	Covance	MMS-435P
mouse anti Map-2 (AP20)	Santa Cruz Biotechnology	sc-32791
rabbit anti Dcx	Abcam	AB18723
mouse anti Nestin	Abcam	ab22035
rabbit anti MeCP2	Cell Signaling	3456S
rabbit anti-AFP	Agilent-DAKO	IR500
rabbit anti-Myf-5	Santa Cruz Biotechnology	sc-302
Alexa Fluor® 488 donkey anti mouse IgG (H+L)	Life Technologies	A21202
Alexa Fluor 555 donkey anti-rabbit IgG (H+L)	Life Technologies	A31572

Supplementary Table S4. List of forward and reverse primers used for qPCR analysis

Gene name	Forward	Reverse	
olig1	CCCCAAAAGTAGCGTAACCA	GCGGTTGGTTTTCGTTTTTA	
gfap	GTGGTGAAGACCGTGGAGAT	CGGAGCAACTATCCTGCTTC	
runx1	CCCTAGGGGATGTTCCAGAT	TGAAGCTTTTCCCTCTTCCA	
brachyury	ACCACCGCTGGAAATATGTGAAC G	AACTCTCACGATGTGAATCCGAG G	
nestin	CAGCGTTGGAACAGAGGTTGG	TGGCACAGGTGTCTCAAGGGTAG	
afp	AGCTTGGTGGTGGATGAAAC	CCCTCTTCAGCAAAGCAGAC	
ncam	ATGGAAACTCTATTAAAGTGAAC CTG	TAGACCTCATACTCAGCATTCCAG T	
olig2	GAAACTACCCCACCGACTCA	ACCCAAACTGTTTCCACAGC	
actin	TGAAGTGTGACGTGGACATC	GGAGGAGCAATGATCTTGAT	
gapdh	ATGGAAATCCCATCACCATCTT	CGG CCC ACT TGA TTT TGG	
tbp	CGGCTGTTTAACTTCGCTTC	CACACGCCAAGAAACAGTGA	

2 Supplementary Figures and Tables

2.1 Supplementary Tables

Supplementary Table S1: Main differential meaningful bands of iPSC-derived NS and EB organoid found in second derivative average spectra and their contribution to PCA analysis. Filled boxes represent contribution of indicated absorbance to the PCA.

Area of second derivative average spectra	Main differential meaningful bands of 3D neural spheres versus EB (cm^{-1})	Main individual absorbances contributing to PCA		Main molecular correspondence	Selected references
		NS vs EB			
Lipid		PC1	PC2		

(2800-3050 cm^{-1})					
	**~2840**				
	**~2850**			v_sCH2	[10, 11]
	**~2875**			v_sCH3	[10, 11]
	**~2890**			*vs(C−H2) cholesterol (in NS)*	[12, 13]
	**~2925**			$v_{as}CH2$	[10, 11]
	**~2960**			$v_{as}CH3$	[10, 11]
	**~3010**			***ν*** *(H−C=) olefinic stretch*	*[14, 15]*
	**~3030**			***ν*** *(H−C=) olefinic stretch*	*[15, 16]*
Protein<br>(1480-1770 cm^{-1})		PC6	PC7		
	**~1515**				
	**~1550**			Amide II α-helix structure and the random coil protein structure	[10, 11, 17, 18]
	**~1570**			Intramolecular β-sheet structure (amide I)	[10, 11, 17, 18]

	**~1590**				
	**~1625**			Intramolecular β-sheet structure (amide I)	[10, 11, 17, 18]
	**~1650**			Random coil structure (amide I) α-helix structure	[10, 11, 17, 18]
	**~1680**			β-turns structure	[10, 11, 17, 18]
	**~1690**			β-sheet structure	[10, 11, 17, 18]
	**~1710**				
	**~1735**			Ester carbonyl groups $\boldsymbol{v}$ (C=O)	[10, 11, 17, 18]
	**~1750**			Ester carbonyl groups (-C=O) in acyl chains of phospholipids	[10, 11, 17, 18]
Nucleic Acid (also phospholipids and phosphorylated proteins) (900 - 1200 cm^{-1})		PC1	PC2		
	**~928**			Z-form DNA	[19-22]
	**~960**				

	**~975**			DNA marker	[19-22]
	**~990**				
	**~1027**			Strongly enhanced in Z-form DNA	[19-22]
	**~1060**			Strongly enhanced in Z-form DNA	[19-22]
	**~1072**			−CO−O−C<br><br>stretching vibrations in cholesterol esters and phospholipids	[10, 13, 17]
	**~1085**			B-form DNA $\boldsymbol{v}$s ($\boldsymbol{PO2}$)	
	**~1110-15**	1115<br>(EB)	1110<br>(NS)	DNA methylation	[19-23]
	**~1150**	1143	1155	vC-O H-bonded carbohydrates (glycocen)	[19-23]
	**~1185**				
	**~1238**				
	**~1263**				

Supplementary Table S2: Integral area mean values of second derivative average spectra of iPSC-derived NS and EB organoid at specific wavelengths ranges related to specific molecules vibration ± standard deviation (SD). The group showing the significant highest integral area mean value for a certain wavelength is labeled in yellow (when NS) or blue (when EB). T-test score, P-value indicating significant (green) or not significant (red) differences between organoids groups.

~ Peak wavelength		Integral Area Mean Value ± SD Primed			
		NS	EB	T-score	P value
~ 930 cm^{-1}	Z-form DNA	-0.010± 0.039	-0.090± 0.0433	7.1	p= 0.0001
~ 990 cm^{-1}	DNA backbone	-0.603± 0.092	-0.504± 0.137	16	p= 0.0001
~ 1060 cm^{-1}	Strongly enhanced z-form DNA	-0.519± 0.074	-0.426± 0.157	13.7	p= 0.0001
~ 1070 cm^{-1}	Cholesterol and phospholipids	-0.120 ± 0.142	-0.1413 ± 0.212	2.2	p= 0.026
~ 112 cm^{-1}	RNA	-0.797± 0.423	-0.407± 0.613	14.2	p= 0.0001
~ 1150 cm^{-1}	vC-O H-bonded carbohydrates	0.34± 0.014	-0.2585± 0.1608	19	p= 0.0001

~ Peak wavelength		Integral Area Mean Value ± SD Primed			
		**NS**	**EB**	**T-score**	**P value**
**~ 1250 cm^{-1}**	**Phospsphate I (vasPO2^{-}) in RNA and phosphorylated molecules. DNA backbone conformation**	-0.073± 0.082	-0.188± 0.177	15	p= 0.0001
**~ 1540 cm^{-1}**	**Amide II**	-2.392± 0.186	-2.522± 0.401	7.5	p=0.0001
**~ 1635 cm^{-1}**	**B-sheet**	-0.659± 0.267	-0.849± 0.352	11.8	p=0.0001
**~ 1650 (1630-1670) cm^{-1}**	**Amide I**	-4.429± 0.514	-4.139± 0.732	8.8	p=0.0001
**~ 1638 cm^{-1}**	**Random coiled (Amide I)**	-0.257± 0.151	-0.327± 0.236	6,6	p=0.0001
**~ 1645 cm^{-1}**	**Random coiled (Amide I)**	-0.812± 0.151	-0.728± 0.186	10	p=0.0001
**~ 1650 cm^{-1}**	**Alpha helix Amide I**	-2.098± 0.280	-1.916± 0.320	12.2	p=0.0001
**~ 1630 cm^{-1}**	**B-structure**	-0.156± 0.036	-0.160± 0.055	1.57	p= 0.115
**~ 1680 cm^{-1}**	**Turns-b**	-0.505± 0.047	-0.052± 0.039	0.7	p= 0.46

~ Peak wavelength		Integral Area Mean Value ± SD Primed			
		NS	EB	T-score	P value
~ 1635/1650 cm^{-1}	B-structure/alpha helix RATIO	0.395± 0.204	0.489 ± 0.528	4.3	p=0.0001
~ 1650/1540 cm^{-1}	Amide I/Amide II (protein structure)	1.930± 0.305	1.826± 0.577	4.5	p=0.0001
~ 1740 cm^{-1}	C=O esther of carbonyl groups (Phospholipids)	-0.108± 0.160	-0.043± 0.176	7.9	p=0.0001
~ 2850 cm^{-1}	sCH2	-1.147± 0.586	-1.522± 0.469	15.8	p=0.0001
~ 2875 cm^{-1}	sCH3	-0.698± 0.402	-0.383± 0.316	19.6	p=0.0001
~ 2922 cm^{-1}	asCH2	-3.661± 0.734	-3.757± 0.488	3.6	p=0.0001
~ 2950 cm^{-1}	asCH3	-0.974± 0.397	-1.137± 0.4787	7.4	p=0.0001
~ 3015 cm^{-1}	Unsaturated fatty acids	-0.083± 0.038	-0.054± 0.045	15	p=0.0001
	CH2 + CH3	-6.134± 0.974	-6.539± 0.836	9.7	p=0.0001
	asCH2 + sCH2 Lipid order/Fluidity/Total lipid	-4.809± 1.15	-5.28± 0.739	16.4	p=0.0001

~ Peak wavelength		Integral Area Mean Value ± SD Primed			
		**NS**	**EB**	**T-score**	**P value**
	**asCH3 + sCH3**	-1.675± 0.629	-1.498± 0.547	6.5	p=0.0001
	**asCH2+asCH3**	-4.635± 0.907	-4.894± 0.809	9.3	p=0.0001
	**sCH2 + sCH3**	-1.624± 0.443	-1.725± 0.382	5.3	p=0.0001
	**sCH2/asCH3 Lipid synthesis**	1.469± 6.837	1.613 ±7.16	0.6	p=0.548
	**sCH2/asCH2 Mb rigidity**	0.301± 0.377	0.391± 0.382	7	p=0.0001
	**asCH2/asCH3 Lipid acyl chain length**	4.153± 1.76	3.833± 1.809	0.7	p=0.465

2.2 Supplementary Figures

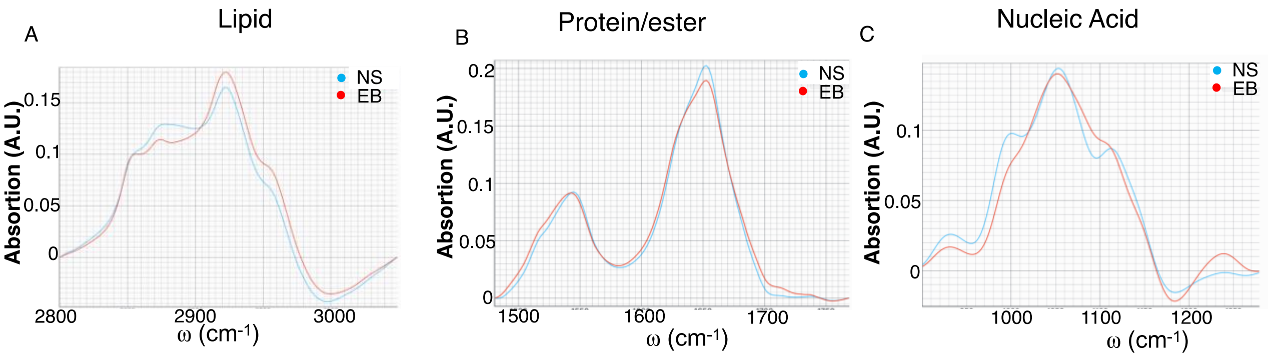

Supplementary Figure S1. FTIR averaged spectra of NS (blue) and EB (red) sectioned organoids in the (A) lipids' spectral region of 3020−2800 cm^{-1}, (B) proteins and carbonyl area, 1800−1480 cm^{-1}, and (C) the nucleic acids region, 1200−900 cm^{-1}

2.3 Supplementary References

1. Lopez-Caraballo, L., et al., *Analysis of Menstrual Blood Stromal Cells Reveals SOX15 Triggers Oocyte-Based Human Cell Reprogramming.* iScience, 2020. **23**(8): p. 101376.
2. Gonzalez-Munoz, E., et al., *Cell reprogramming. Histone chaperone ASF1A is required for maintenance of pluripotency and cellular reprogramming.* Science, 2014. **345**(6198): p. 822-5.
3. Lopez-Caraballo, L., et al., *iPS-Derived Early Oligodendrocyte Progenitor Cells from SPMS Patients Reveal Deficient In Vitro Cell Migration Stimulation.* Cells, 2020. **9**(8).
4. Marton, R.M., et al., *Differentiation and maturation of oligodendrocytes in human three-dimensional neural cultures.* Nat Neurosci, 2019. **22**(3): p. 484-491.
5. Shihan, M.H., et al., *A simple method for quantitating confocal fluorescent images.* Biochem Biophys Rep, 2021. **25**: p. 100916.
6. Schindelin, J., et al., *Fiji: an open-source platform for biological-image analysis.* Nat Methods, 2012. **9**(7): p. 676-82.
7. Demšar J, C.T., Erjavec A, Gorup Č, Hocevar T, Milutinovič M, Martin Možina M, Polajnar M, Toplak M, Starič A, Štajdohar M, Umek L, Žagar L, Žbontar J, Žitnik M, Zupan B. , *Orange: data mining toolbox in Python.* J Mach Learn Res 2013. **14**: p. 2349–2353.
8. Toplak M., B.G., Read S., Sandt C., Rosendahl S.M., Vaccari L., Demšar J., Borondics F., *Infrared Orange: Connecting Hyperspectral Data with Machine Learning.* Synchrotron Radiat. News., 2017. **30**: p. 40-45.
9. Toplak, M., et al., *Quasar: Easy Machine Learning for Biospectroscopy.* Cells, 2021. **10**(9).

10. Loutherback, K., et al., *Microfluidic approaches to synchrotron radiation-based Fourier transform infrared (SR-FTIR) spectral microscopy of living biosystems.* Protein Pept Lett, 2016. **23**(3): p. 273-82.
11. Martinez-Rovira, I., et al., *Study of the intracellular nanoparticle-based radiosensitization mechanisms in F98 glioma cells treated with charged particle therapy through synchrotron-based infrared microspectroscopy.* Analyst, 2020. **145**(6): p. 2345-2356.
12. Sandt, C., J. Frederick, and P. Dumas, *Profiling pluripotent stem cells and organelles using synchrotron radiation infrared microspectroscopy.* J Biophotonics, 2013. **6**(1): p. 60-72.
13. Dučić, T., et al., *Live-Cell Synchrotron-Based FTIR Evaluation of Metabolic Compounds in Brain Glioblastoma Cell Lines after Riluzole Treatment.* Anal Chem, 2022. **94**(4): p. 1932-1940.
14. Giorgini, E., et al., *Vibrational characterization of female gametes: a comparative study.* Analyst, 2014. **139**(20): p. 5049-60.
15. Malek, K.W., B. R.; Bambery, K. R, *FTIR Imaging of Tissues: Techniques and Methods of Analysis. In Optical Spectroscopy and Computational Methods in Biology and Medicine.* Springer Netherlands, 2014: p. 419-473.
16. Karimi, F., et al., *Infrared microspectroscopy studies on the protective effect of curcumin coated gold nanoparticles against H2O2-induced oxidative stress in human neuroblastoma SK-N-SH cells.* Analyst, 2021. **146**(22): p. 6902-6916.
17. Dučić, T., et al., *Multimodal Synchrotron Radiation Microscopy of Intact Astrocytes from the hSOD1 G93A Rat Model of Amyotrophic Lateral Sclerosis.* Anal Chem, 2019. **91**(2): p. 1460-1471.
18. Heraud, P., et al., *Fourier transform infrared microspectroscopy identifies early lineage commitment in differentiating human embryonic stem cells.* Stem Cell Res, 2010. **4**(2): p. 140-7.
19. Zhang, F., et al., *Histone Acetylation Induced Transformation of B-DNA to Z-DNA in Cells Probed through FT-IR Spectroscopy.* Anal Chem, 2016. **88**(8): p. 4179-82.
20. Kim, S.H., et al., *Unveiling the pathway to Z-DNA in the protein-induced B-Z transition.* Nucleic Acids Res, 2018. **46**(8): p. 4129-4137.
21. Li L., L.S.F., Puretzky A., Riehn R., Hallen H.D, *DNA Methylation Detection Using Resonance and Nanobowtie-Antenna-Enhanced Raman Spectroscopy.* Biophysical Journal, 2018. **114**(11): p. 2498.
22. Dučić, T., et al., *Monitoring oocyte-based human pluripotency acquisition using synchrotron-based FTIR microspectroscopy reveals specific biomolecular trajectories.* Spectrochim Acta A Mol Biomol Spectrosc, 2023. **297**: p. 122713.
23. Gioacchini, G., et al., *A new approach to evaluate aging effects on human oocytes: Fourier transform infrared imaging spectroscopy study.* Fertil Steril, 2014. **101**(1): p. 120-7.
